# An Experimental and Numerical Study on the Mechanical Properties and Damage Evolution of Cemented Tailings Backfill Under Uniaxial Compression

**DOI:** 10.3390/ma18040856

**Published:** 2025-02-15

**Authors:** Congxiang Yuan, Houqiang Wang, Zhixiang Liu, Shuangxia Zhang, Mengyang Yan, Xiaodie Liang, Zhiwei Liu, Weijun Liu

**Affiliations:** School of Resources and Safety Engineering, Central South University, Changsha 410083, China; yuancx77@163.com (C.Y.); w13015388206@gmail.com (H.W.); 205507032@csu.edu.cn (S.Z.); yanmengyang8@163.com (M.Y.); 225511026@csu.edu.cn (X.L.); 225511028@csu.edu.cn (Z.L.); 225501013@csu.edu.cn (W.L.)

**Keywords:** cemented tailings backfill, mechanical properties, numerical simulation, damage constitutive model, engineering application

## Abstract

A comprehensive understanding of the mechanical behavior of backfill under compression is crucial for optimizing its design, improving stope stability and enhancing resource recovery. Laboratory testing and numerical simulation were conducted to study the mechanical properties and damage mechanism of cemented tailings backfill (CTB) with different cement-to-tailings (c/t) ratios under uniaxial compression. Laboratory testing was used to investigate the strength and deformation characteristics, macroscopic failure modes, and energy evolution patterns of CTB, while simulation with Particle Flow Code (PFC) was employed to explore the distribution of microcracks and mesoscopic damage mechanisms. A constitutive model accounting for the initial compaction stage was proposed, validated, and applied to practical engineering. The results show that as the c/t ratio decreases, the failure mode of CTB transforms from shear failure to combined tensile–shear failure, and tensile failure. Mesoscopically, a higher c/t ratio leads to more bond contacts, which increases the bearing capacity and consequently causes more cracks to damage CTB. From an energy standpoint, the damage mechanism of CTB is further analyzed and the development of energy is characterized by four stages. Moreover, to explore the failure mechanism of CTB, an innovative constitutive model was proposed and verified through experiments. The matching coefficients, based on the novel constitutive model, indicate that CTB with a c/t ratio of 1:6 is qualified for all current mining depths, and a c/t ratio of 1:10 is sufficient to depths below 300 m.

## 1. Introduction

Mining, as a traditional industry, contributes to driving socio-economic development and meeting the increasing demand for mineral resources [1,2,3,4]. However, irrational resource extraction usually leads to significant wastage of mineral resources, jeopardizes safety in production and even causes severe environmental impacts. The massive accumulation of metal-containing waste rock from mining operations in tailings ponds and dams not only occupies and degrades extensive land resources but also increases risks such as dam failures and instability, worsening ecological degradation [5,6,7,8,9,10]. Furthermore, with shallow mineral resources becoming scare, deep mining has become the norm, facing heightened challenges of three “high” and one “disturbance” [11,12,13]. To have these issues disposed effectively, filling mining, especially CTB mining, is usually prioritized. This approach not only utilizes tailings efficiently, making beneficial use of waste, but also mitigates the challenges associated with deep mining, making backfill mining a means for economic growth and a pivotal pathway towards sustainable development [14,15,16,17,18,19,20].

Consequently, CTB has attracted much attention from scholars worldwide and been widely applied in filling technology. Usually, dewatered tailings are utilized as aggregates and cement as a binder by adding other additives to make CTB. Numerous studies on CTB incorporating different aggregates, c/t ratios and additives were conducted to examine physical mechanical properties and failure modes through laboratory testing and numerical simulation [21,22,23]. For example, Nasir and Fall [24] developed a numerical model considering the coupled thermo-chemical loadings to forecast the uniaxial compressive strength (UCS) of undrained cemented paste backfill (CPB) and validated it against experimental results. Ghirian and Fall [25,26] investigated the coupling thermo–hydro–mechanical–chemical behavior of CPB to reveal the evolution of these processes through column experiments. Yin et al. [27] found that there was a significant relationship between macroscopic strength and texture features that embodied the microstructure at different solid concentrations of the backfill specimen by testing strength and extracting scanning electron microscope (SEM) images. Mangane et al. [23] explored the influences of five superplastic admixtures at varying doses on the mechanical characteristics and workability of CTB.

Further, the energy evolution, microcrack development, and damage evolvement characteristics of CTB were studied to reveal the energy distribution and interpret the microcrack extension mechanism and damage accumulation under external loading, contributing to the optimization and design of CTB. Zhao et al. [28] explored the meso-mechanism of the coupled effect of waste rock and initial damage on the mechanical responses of cemented tailings-rock backfill by PFC. Yin et al. [29] investigated the mechanical properties, energy evolution, and failure patterns of fiber-reinforced cemented sulfur tailings backfill and established a constitutive model incorporating the fiber factor, the damage correction coefficient, and the damage threshold under uniaxial loading. Gan et al. [30] studied the energy evolution characteristics and created a constitutive model of early-age CPB under cyclic load.

The studies mentioned above lay a solid theoretical foundation for understanding the role of CTB in supporting and maintaining the stability of goafs. However, as an artificial composite material, CTB develops micropores and microcracks during preparation, transportation, and hardening in engineering, resulting in microdefects. And in the laboratory, the micropores and microcracks occur and are compacted during the initial stage under compression. Therefore, classical damage constitutive models fail to accurately simulate this stage, resulting in the irrationality of energy matching with surrounding rocks in engineering.

Findings show that these microdefects cause initial damage and inform the damage mechanism of the microstructure [31,32,33,34,35], giving the compaction property of CTB. Consequently, the constitutive model considering initial defects has intrigued domestic and foreign scholars, and numerous related studies have been published. Liu et al. [36] proposed a segmental damage constitutive equation for cemented coal gangue–fly ash backfill at varying maintenance temperatures considering the initial compaction phase to describe damage evolution. Wang et al. [7] developed a new CPB by adopting electrolytic manganese residue, red mud and slag as binders and established a damage intrinsic model, which incorporated the initial compaction-density stage. Zhao et al. [34] analyzed the early macroscopic mechanical properties of cemented superfine tailings backfill containing various initial defects, and established a coupled damage equation incorporating both initial damage and loading-induced damage. However, axial crack evolution and the constitutive model for CTB from this view have been scarcely concerned.

By selecting a suitable constitutive model, the mechanical properties of CTB can be more accurately reproduced and predicted, thereby improving the safety and reliability of engineering design and construction. This paper systematically studied the macroscopic mechanical properties, stress–strain curves, failure modes, distribution characteristics of microcracks and energy evolution patterns with methods of laboratory testing and numerical simulation. Furthermore, a damage constitutive model considering the initial compaction stage incorporating the axial crack evolution was established and verified against experimental data. Using the novel constitutive model and energy matching principle, the deformation energy of CTB and the matching coefficients for varying c/t ratios and mining depths were calculated to explore the practical and economic application of CTB in the 5# ore body of Chaihulanzi Gold Mine.

## 2. Materials and Methods

### 2.1. Materials

**Figure 1:**
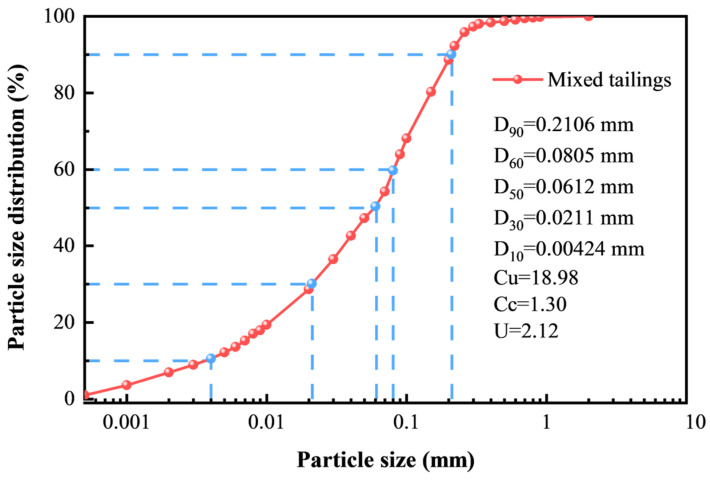
Size distribution curve of tailings.

**Figure 2:**
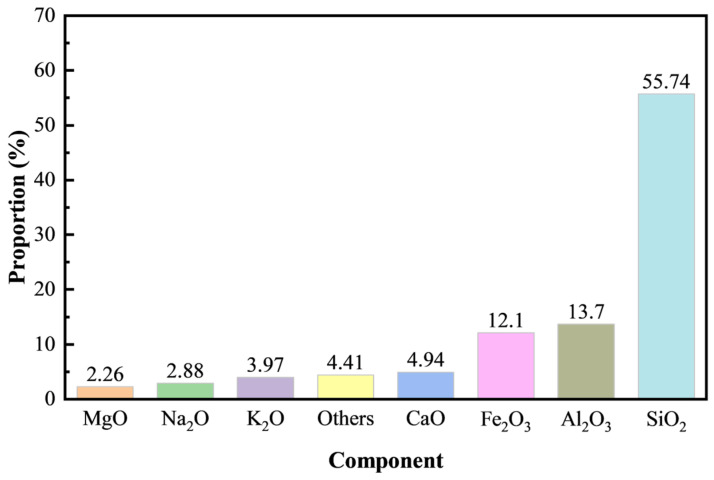
Chemical composition of tailings.

The tailings for aggregate used in this test were sampling at a tailing reservoir at a metal mine in Yunnan, China. A laser particle sizer was adopted to determine the particle size distribution and the curve of mixed tailings is shown in Figure 1. *D*_10_, *D*_30_, *D*_50_, *D*_60_ and *D*_90_ represent the particle size of the cumulative mass proportion with 10%, 30%, 50%, 60% and 90%. As shown in Figure 1, the uniformity coefficient *C*u (*C*u = *D*_60_/*D*_10_), the curvature coefficient *C*c (*C*c = *D*^2^_30_/*D*_60_ × *D*_10_) and indexing uniformity *U* are 18.98, 1.30, and 2.12, respectively, indicating a uniform classification. The chemical composition of mixed tailings was analyzed by X-ray fluorescence (XRF), as shown in Figure 2. The content of SiO_2_ is 55.74% as the highest proportion and the rest were mainly metallic oxides. The cemented material utilized was PO.42.5R cement, which followed the Chinese Standard GB 175-2007 [37]. Tap water in the laboratory was used to mix the raw materials.

### 2.2. Experimental Scheme and Sample Preparation

**Figure 3:**
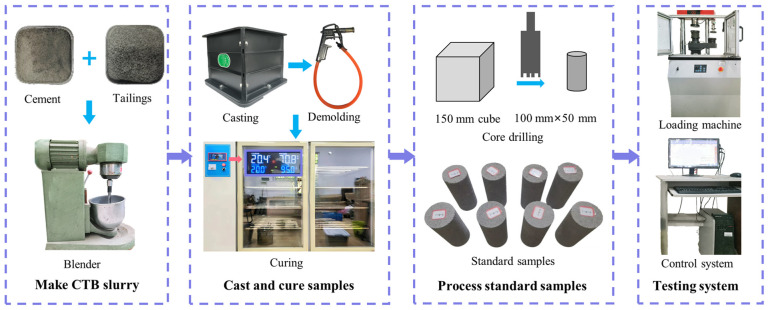
CTB sample preparation and uniaxial compression tests.

The CTB samples were processed with c/t ratios of 1:4, 1:6, 1:8 and 1:10, respectively, and a slurry solid mass concentration of 78%. The CTB sample preparation process is elucidated as follows and shown in Figure 3.

(1) Make CTB slurry: The tailings were placed in an oven for drying to remove the moisture. The tailings and cement were weighed in sequence following the designed maximum ratios aforementioned and mixed evenly in a blender. To ensure that the slurry was uniformly mixed, the mixture was stirred for at least 3 min after quantified water was added.
(2) Cast and cure samples: Before pouring, Vaseline is applied to the molds to facilitate demolding. The slurry was poured into 150 mm × 150 mm × 150 mm cubic molds and vibrated for 2 min on a shake table to smooth out bubbles. After 24 h, the cubes were demolded with demolding gun and kept in a curing box for 28 d with a constant temperature of 20 ± 2 °C and humidity of 95 ± 5%.
(3) Process standard samples: The cubes were sampled with a core drilling machine and the cylindrical samples were polished to standard dimensions of 50 mm in diameter and 100 mm in height.

### 2.3. The Testing System and Methods

To reduce the discreteness, samples with obvious cracks and incomplete samples after processing were discard. And the wave velocity tester was employed to select 3~5 samples with similar wave velocities and densities of each set. The test device was the WHY-300/10 microcomputer-controlled pressure testing machine, manufactured by Shanghai Hualong Test Instruments Corporation (Shanghai, China)., which was composed of a control system, a monitor, a loading machine and a displacement extensometer monitoring device, as shown in Figure 3. Standard CTB samples underwent uniaxial compression tests to obtain the mechanical properties at a loading rate of 0.20 mm/min. The average values were selected as the final test results.

### 2.4. Numerical Simulation

Laboratory testing intuitively reflects the macroscopic mechanical properties and damage modes of CTB, Nevertheless, the crack extension and microscopic damage mechanisms cannot be visualized and investigated from a micro perspective [38,39]. Therefore, the discrete element simulation program, PFC^2D^ 5.0, was employed to make up for this deficiency, and to corroborate the laboratory results.

**Figure 4:**
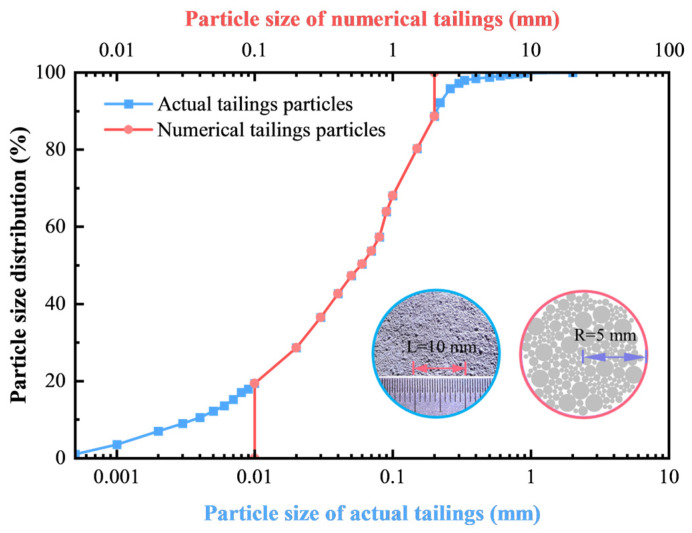
Particle size distribution of actual and numerical tailings.

Given the specificities of CTB, including the varying particle sizes of tailings and the cementation effect from cement hydration, two groups of particles were used to construct the CTB model. One group was tailings particles, while the other group was cementitious particles, which represent the hydration products of cement in CTB. This approach was based on the CTB model developed by Liu et al. [40]. To simulate the actual situation, numerical models with dimensions of 50 mm × 100 mm were established. The models included tailings particles with specific grading and porosity, and certain numbers of cementing particles. For simulation, this paper simplified the particle size distribution curve by ignoring the start and end parts of it and replacing the ignored data with the corresponding maximum and minimum particle sizes of 0.1 mm and 1.5 mm, respectively [40,41]. Simultaneously, to save computational time without affecting the computational accuracy, the particle gradation was adjusted by up-scaling the particle radius by 10 folds, which was verified to be reasonable and employed in numerical simulations [42,43,44,45]. Meanwhile, the cementitious particles were slightly finer than the smallest tailings particles, which were 0.1 mm in diameter. Figure 4 shows the particle size distribution curves and comparison of actual tailings and processed numerical tailings.

**Figure 5:**
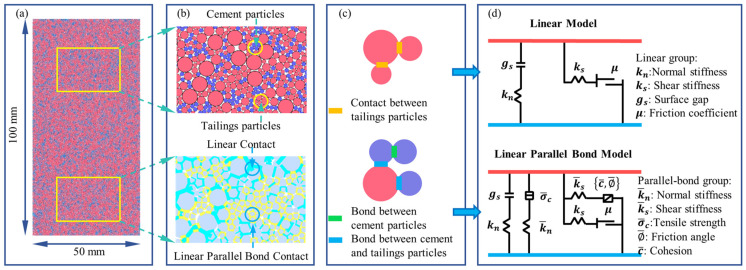
Establishment of the numerical simulation model of CTB: (**a**) the CTB sample; (**b**) particle and contact groups; (**c**) contact assignment; (**d**) contact models.

The parallel bond contact model (PBM) was employed to generate bonds between assigned particles, allowing force and moment to be transferred between bonded particles [45,46]. The PBM was primarily assigned to the contact between cement particles and adjacent particles, while linear contact was formed between tailings particles themselves (Figure 5b). The progress could be realized by using the command of “contact groupbehavior and”, which allows users to specify the behavior of filtering contacts by specific groups, as shown in Figure 5c. Parallel bonding refers to a certain thickness of bonding material between contacting particles. When the parallel bond contact fails, the forces and moments acting on the parallel bond contact in the calculation will become zero, and the PBM degenerates into a linear model (Figure 5d). This modeling method can accurately reflect the microstructure of CTB material, and has been applied by many scholars to study the meso-scale damage characteristics and mechanical responses of CTB [40,47,48].

The uniaxial compression process was numerically performed by applying velocities to the upper and bottom walls as servo-controlled boundary stresses. The walls moved at a specified speed of 0.2 mm/min until the post-peak axial stress decreased to 50% of the peak value. Meanwhile, the loading process was recorded using the built-in Fish language to generate the stress–strain curve, and capture the type, location, and number of microcracks.

## 3. Results and Discussion

### 3.1. Macroscopic Mechanical Properties

**Figure 6:**
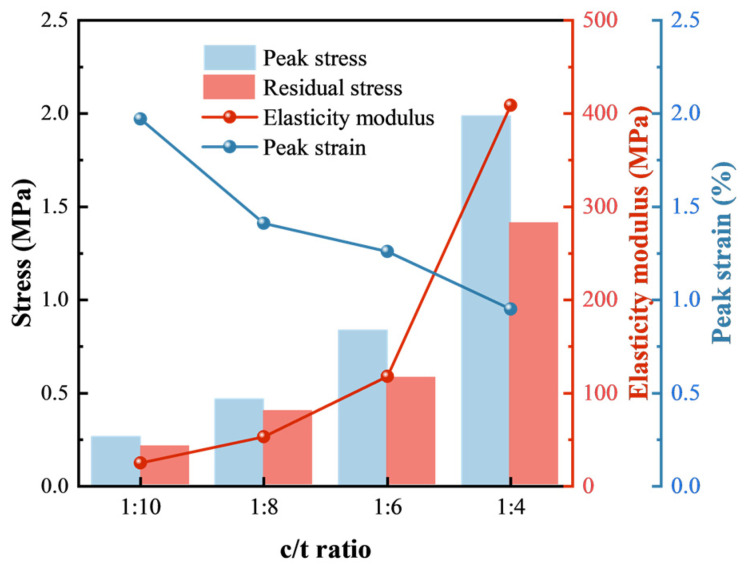
Mechanical properties of CTB with different c/t ratios.

Studying strength and deformation characteristics, such as the peak stress, the residual stress, the elasticity modulus, and the peak strain, is essential for understanding the mechanical behavior of CTB, which reflect its stress limit and compressibility. The mechanical characteristics were determined by solving the mean of experimental data in each set. Figure 6 shows the change in the four characteristics of CTB with different c/t ratios.

#### 3.1.1. Analysis of Strength Characteristics

**Figure 7:**
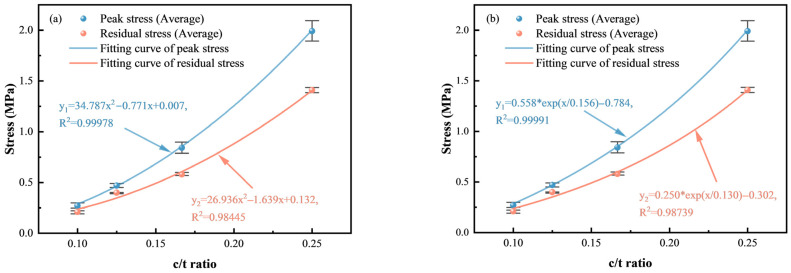
Relationship between the peak stress, residual stress and c/t ratios: (**a**) polynomial function fitting; (**b**) exponential function fitting.

Figure 6 illustrates that both the peak stress and the residual stress increase as the c/t ratio increases. The highest values are observed at a c/t ratio of 1:4, with peak and residual stresses of 1.99 MPa and 1.41 MPa, respectively. At lower c/t ratios of 1:6, 1:8, and 1:10, the peak stress decreases by 57.8%, 76.4%, and 86.4%, respectively, while the residual stress decreases by 58.9%, 71.6%, and 85.1%, respectively. Obviously, the relationships between the c/t ratio and both the peak stress and the residual stress are not simply linearly increasing but instead conforming to the change patterns of polynomial and exponential functions. Therefore, they are adopted to describe the relationship of these stresses of CTB and different c/t ratios according to the experimental results, as shown in Figure 7. The correlation coefficients (*R*^2^) for polynomial and exponential fits of the peak stress and residual stress are 0.99978, 0.98739, 0.98445 and 0.99991, respectively. Hence, exponential or polynomial functions could be adopted to describe how the peak stress and residual stress vary with the c/t ratio, as follows:

(1) $$\sigma_{p}=\sigma_{p}\left(x\right)$$

(2) $$\sigma_{r}=\sigma_{r}\left(x\right)$$

where $\sigma_{p}$ is peak strength, $\sigma_{r}$ is the residual stress, and *x* is the c/t ratio.

#### 3.1.2. Analysis of Deformation Characteristics

**Figure 8:**
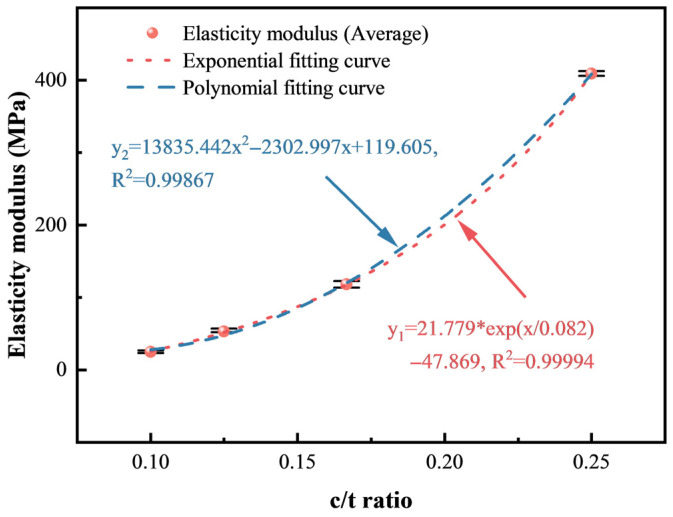
Relationship between the elasticity modulus and the c/t ratio.

**Figure 9:**
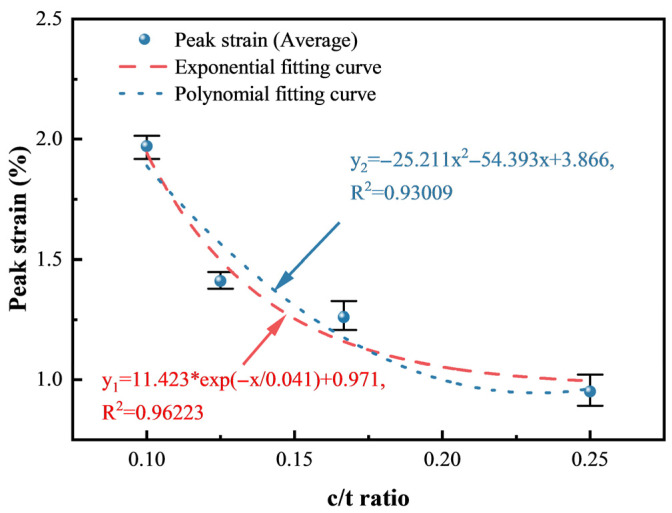
Relationship between the peak strain and the c/t ratio.

Compared to CTB with a c/t ratio of 1:4, the elasticity modulus with c/t ratios of 1:6, 1:8, and 1:10 decreases by 71.1%, 87.0% and 93.9%, respectively. The peak strain increases by 32.6%, 48.4%, and 107.4%, respectively. Similarly, polynomial and exponential functions are employed to describe how the peak stress and residual stress change with the c/t ratio, as shown in Figure 8 and Figure 9. The *R*^2^ values of polynomial fitting and exponential fitting for the elasticity modulus are 0.99867 and 0.99994, respectively, while the values for the peak strain are 0.93009, and 0.96233, respectively. The *R*^2^ values of the fitting curves all exceed 0.9, elucidating a strong polynomial or exponential relationship between the elasticity modulus and the c/t ratio. Similarly, the peak strain also shows a strong relationship with the c/t ratio.

(3) $$E=E\left(x\right)$$

(4) $$\varepsilon_{p}=\varepsilon_{p}\left(x\right)$$

where *E* is the elasticity modulus and $\varepsilon_{p}$ is the peak strain.

Error bars serve as a visual representation of data variability and uncertainty. As depicted in Figure 7, Figure 8 and Figure 9, shorter error bars indicate that data points are clustered around the mean value, suggesting lower variability and more precise measurements. Consequently, utilizing the average for data fitting is a justified approach, as it better reflects the central tendency of the data while mitigating the impact of variability on the model fitting results. Table 1 shows that the average *R*^2^ values for polynomial and exponential function fittings of different mechanical parameters are 0.97825 and 0.98739, respectively. Therefore, exponential function fitting can better describe the relationships of the mechanical parameters and the c/t ratio.

### 3.2. Stress–Strain Curves

#### 3.2.1. Experiment Results

**Figure 10:**
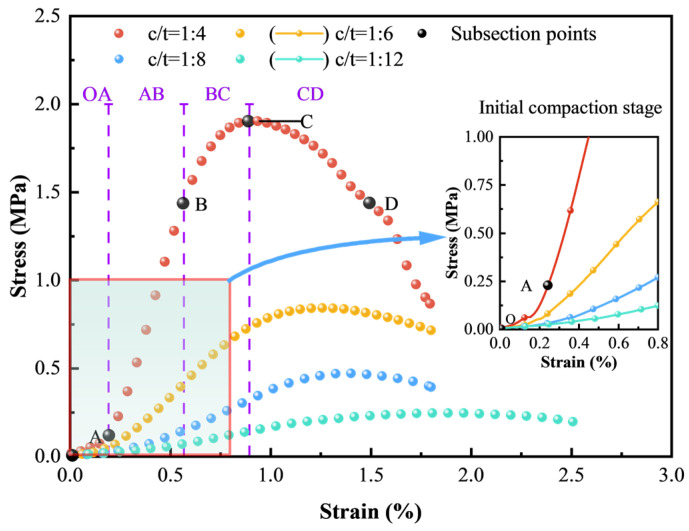
Stress–strain curves of CTB with different c/t ratios.

The stress–strain curve is crucial for analyzing the mechanical properties of CTB, providing insights into its deformation and failure behaviors under different stress conditions. Figure 10 illustrates that the stress–strain curves drawn with experimental data of CTB with different c/t ratios exhibit striking similarities. According to the deformation characteristics and curve patterns, stress–strain curves can be divided into four stages, and the curve of a c/t ratio of 1:4 is selected to demonstrate. These stages are: the initial compaction stage (OA), the elastic stage (AB), the plastic stage (BC), and the post-peak stage (CD). Notably, there is an obvious compaction characteristic and CTB with different c/t ratios display varying deformation characteristics. For CTB with a lower c/t ratio, the compaction stage is more pronounced due to the existence and increased quantity of microdefects.

#### 3.2.2. Meso-Parameters Calibration and Simulation Results

**Figure 11:**
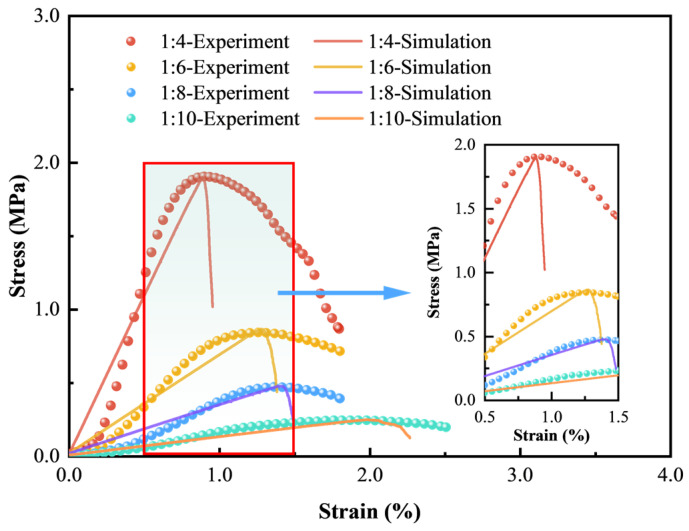
Comparison of stress–strain curves between experiment and simulation results.

The trial-and-error method was adopted to calibrate the meso-parameters of the simulation model until the stress–strain curves matched well with the laboratory results and the macro failure modes were similar. The calibrated meso-parameters of samples with four c/t ratios are summarized in Table 2. Figure 11 presents the stress–strain curves from numerical simulations compared to laboratory tests. Although there are some deviations in the initial compaction stage owing to the absence of micropores in simulation models compared to actual samples, the stress–strain curves are roughly overlapping, especially regarding the peak stress and the peak strain. The simulation results correspond well with experiment data, validating the reasonableness of the established CTB models.

### 3.3. Macroscopic Failure Modes and Distribution Characteristics of Microcracks

#### 3.3.1. Macroscopic Failure Modes

**Figure 12:**
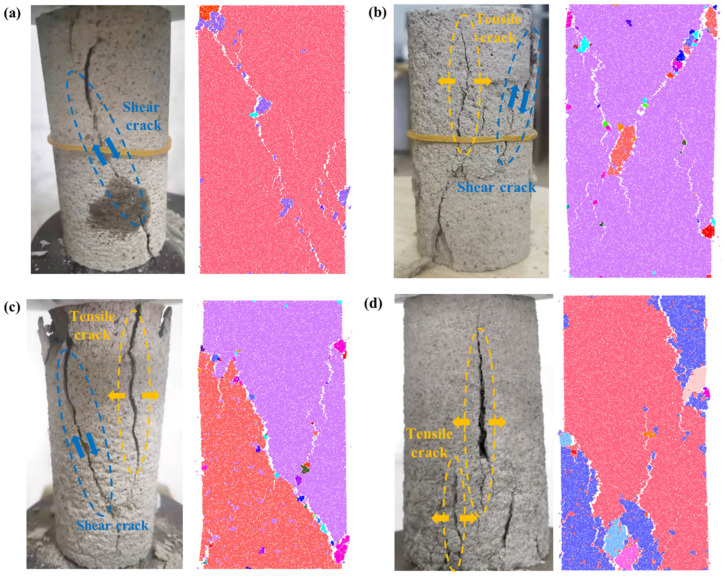
Failure modes: (**a**) c/t = 1:4; (**b**) c/t = 1:6; (**c**) c/t = 1:8; (**d**) c/t = 1:10.

Analyzing the failure characteristics and modes is vital for clarifying the damage mechanism of CTB. There are usually three typical failure modes under uniaxial compression on CTB. Figure 12 shows the failure modes and macro fracture distribution observed in experiments and simulations. The failure mode of CTB transforms from shear failure to combined tensile–shear failure, and tensile failure. Further, the damage patterns observed in the simulations broadly align with those recorded in experiments. According to Figure 12a, the failure mode of CTB basically conforms to the characteristics of monoclinic shear failure. After failure, a penetrating crack forms at an angle of approximately 45° to the loading direction, indicating shear failure. For CTB with a c/t ratio of 1:6, Figure 12b shows that two large macro fractures develop—one generally aligns with the direction of loading, while the other extends at approximately a 50° angle to the horizontal plane. Consequently, this results in the sample experiencing both shear and tensile stresses, exhibiting a mixed tensile–shear failure. The failure mode of CTB with a c/t ratio of 1:8 shows a similar pattern to that with a c/t ratio of 1:6, dominated by one vertical fracture and one inclined fracture, as shown in Figure 12c. Conversely, CTB with a c/t ratio of 1:10 (Figure 12d) primarily exhibits vertical macro tensile fractures because of the Poisson effect, where the sample undergoes lateral tensile failure due to lateral tensile stress surpassing the sample’s tensile strength.

#### 3.3.2. Distribution and Evolution Characteristics of Microcracks

**Figure 13:**
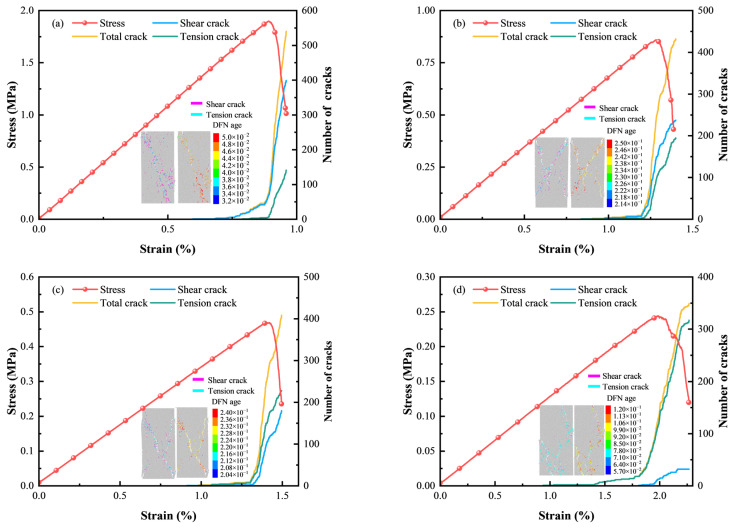
Microcrack number, evolution and distribution: (**a**) c/t = 1:4; (**b**) c/t = 1:6; (**c**) c/t = 1:8; (**d**) c/t = 1:10.

Laboratory testing can only obtain the evolution of macrocracks and final failure modes superficially, making it difficult to reveal the initiation, extension, and penetration of microcracks. However, numerical simulation can offset this limitation and provide insight into the meso-damage mechanism of CTB by reproducing the evolution of microcracks. In simulation, microcracks propagate, merge, and converge into macro fractures as parallel bonds break under external loading. The evolution and number of microcracks are portrayed in Figure 13, indicating shear macrocracks with magenta lines and tension microcracks with cyan lines. Additionally, the distribution of microcracks and the age evolution of discrete fracture network (DFN) are also plotted in Figure 13 to provide further details on the meso-damage process.

From the perspective of crack number, the total microcracks number increases as the c/t ratio increases, mainly because a higher c/t ratio leads to more generated bond contacts, making crack initiation more difficult and enhancing the material’s resistance to failure. Further, with a reduction in the c/t ratio, the number of shear microcracks decreases while the number of tension microcracks increases. Regarding microcrack distribution, shear cracks develop along a bevel at a 45° angle with the radial plane until they perforate CTB, creating a leading macro fracture, while tension cracks are rare, as shown in Figure 13a. Figure 13b shows that CTB forms a macroscopic primary fracture and generates shear cracks followed by tension cracks along the primary fracture in the loading direction. According to Figure 13c, CTB with a c/t ratio of 1:8 exhibits similar microcrack propagation as CTB with a c/t ratio of 1:6, but has more tensile microcracks and fewer shear microcracks. In contrast, CTB with a c/t ratio of 1:10 (Figure 13d) finally forms a macroscopic tensile fracture that is almost parallel to the loading direction. A fracture property, DFN age, is determined as the accumulated age and records along with the fracture quantity and distribution. DFN age is shown in Figure 13 to uncover the microcrack evolution process. In Figure 13a–c, it is obvious that microcracks appear from one end of the sample, propagate along a single inclined plane, and penetrate the sample. In Figure 13d, microcracks occur and propagate inside, leading to the sample destruct, and there is microcrack aggregation in local scopes of CTB because of the Poisson effect. Changes in microcracks number and distribution of microcracks with varying c/t ratios suggest that cement content could influence the stress distribution in CTB. This is because variations in the c/t ratio alter the mechanical properties (such as strength and brittleness) and microstructure (particles arrangement and contact bonds distribution). CTB with a higher c/t ratio is prone to shear failure in stress concentration areas as microcracks tend to propagate in the direction of maximum shear stress under uniaxial compression.

### 3.4. Energy Evolution Patterns

From an energy perspective, CTB receives energy input from the loading machine under external loads, which is gradually transformed into elastic strain energy and stored in CTB. Then, with continuous loading, energy is released and dissipated, leading to CTB failure. The calculation formulas of energy transformation of CTB under compression loads can be expressed as [49,50]:

(5) $$U=U_{d}+U_{e}$$

(6) $$U=\int \sigma_{1}d\varepsilon_{1}$$

(7) $$U_{e}=\frac{{\sigma_{1}}^{2}}{2E}$$

where U, $U_{d}$ and $U_{e}$ represent, respectively, the total energy, dissipated energy, and elastic strain energy under a one-dimensional stress state; $\varepsilon_{1}$ is axial strain; and $\sigma_{1}$ is axial stress.

**Figure 14:**
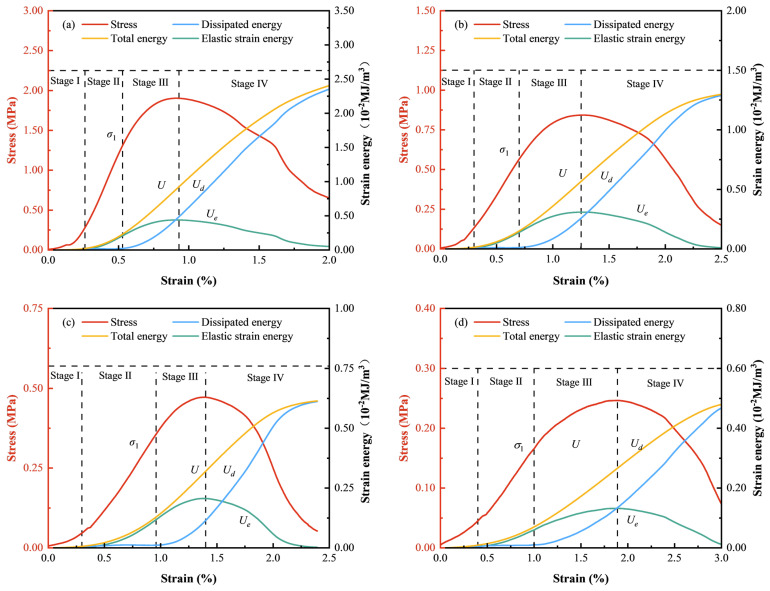
Energy evolution characteristics: (**a**) c/t = 1:4; (**b**) c/t = 1:6; (**c**) c/t = 1:8; (**d**) c/t = 1:10.

Figure 14 indicates the energy evolution of CTB with different c/t ratios under uniaxial compressive loading. The energy evolution patterns of four specimens are broadly similar and can be divided into four stages according to the shape and variation trend of the stress–strain curves: initial compaction, elastic, plastic, and failure stages.

(1) During the first stage, the slope of the stress–strain curve increases slowly. The external load energy is converted into total energy to compress the microdefects within CTB. At this stage, elastic strain energy is nearly equal to total energy, with dissipated energy approaching zero. (2) In the second stage, the curves for total energy and elastic strain energy change from almost overlapping to separating, with dissipated energy increasing slightly. This indicates that almost all the input energy is converted into elastic strain energy and stored in CTB, causing a rapid accumulation of elastic strain energy. (3) As the stress–strain curve enters the third stage, it becomes convex. The stress on CTB reaches its elastic limit, and the rate of elastic strain energy accumulation decreases with increasing external loads. During this stage, CTB begins to become damage, as the dissipated energy accumulates rapidly with a downward concave curve shape, though it remains lower than the accumulated elastic strain energy. At the peak stress, the elastic strain energy decreases with the reduction in the c/t ratio. However, the proportion of dissipated energy to total energy exhibits a decreasing trend, indicating that CTB with a higher c/t ratio undergoes a significant amount of plastic deformation prior to failure. (4) When the stress peaks, the accumulation of elastic strain energy within CTB peaks and begins to release, causing the elastic strain energy curve to decline gently. Meanwhile, dissipated energy accumulates sharply in a linear fashion first and slows down later, with damage continuing to accumulate until CTB completely fails. At this phase, the elastic strain energy of CTB approaches zero, showing that most of the input energy is released as dissipated energy. For CTB with a lower c/t ratio, a greater axial strain (deformation) is required to inhabit the transition from elastic strain energy into dissipated energy. Consequently, with strain increasing, the elastic strain energy initially increases slowly, then dramatically, and eventually decreases slowly, and the dissipated energy increases in a “gentle–rapid–steady–slow” pattern.

### 3.5. Analysis of Axial Crack Evolution

The stress–strain curve of CTB, as depicted in Figure 10, exhibits a distinct compaction stage. The stage is particularly pronounced at a lower cement content and is notably prominent in CTB with a c/t ratio of 1:10. The compaction stage primarily arises from microdefects formed during the casting and hardening process. To investigate the evolution law of these microdefects, axial crack strain is introduced to analyze the deformation of microdefects within CTB. Axial crack strain refers to the axial deformation resulting from the initiation and propagation of initial microdefects and the formation of new microdefects under external loads [51,52,53]. In uniaxial compression, axial crack strain equals axial strain minus elastic deformation [54]:

(8) $${\varepsilon_{1}}^{c}=\varepsilon_{1}-\frac{\sigma_{1}}{E}$$

where ${\varepsilon_{1}}^{c}$ and $\varepsilon_{1}$ are axial crack strain and axial strain, respectively.

**Figure 15:**
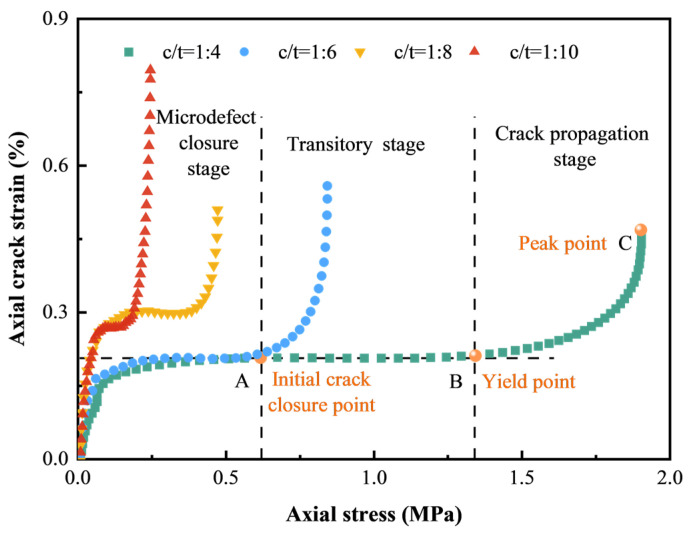
Axial crack strain versus axial stress curves of CTB with different c/t ratios.

Figure 15 depicts axial crack stress–strain curves for CTB with varying c/t ratios, illustrating a consistent trend. For instance, the curve of CTB with a c/t ratio of 1:4 corresponds to three pre-peak stages in the stress–strain curve. According to the shape and variation trend of both curves, the axial crack stress–strain curve can be divided into three obvious stages: microdefect closure, transitory (stabilization), and crack propagation. These stages elucidate the compaction, closure, development, and penetration of microcracks within CTB. During the microcrack closure stage, axial crack strain initiates due to initial microdefects. Under initial loading, the existence of the most initial microdefects results in a fast increase in axial crack strain with a steep curve slope. As these initial microdefects gradually close, the number of microdefects decreases, leading to a diminished rate of axial crack strain growth. In the transitory stage, nearly all initial microdefects are closed, and no new microdefects emerge, resulting in a nearly stable axial crack strain with a slope approaching zero. Upon reaching the yield point, as CTB enters the plastic stage, new microdefects generate under increasing external loads, causing a change in the curve shape from horizontal to concave–upward. As axial stress is exerted further, microdefects gradually grow and extend, causing the curve slope to approach infinity in the crack propagation stage. Additionally, as the c/t ratio increases, the transitory (stabilization) stage becomes more pronounced. For CTB with a lower c/t ratio, such as 1:10, the elastic stage is comparatively brief. This is due to a higher initial microdefect content and lower strength, facilitating quicker attainment of the yield point. When the axial stress peaks, the microcracks further develop and penetrate, causing CTB to fail.

**Figure 16:**
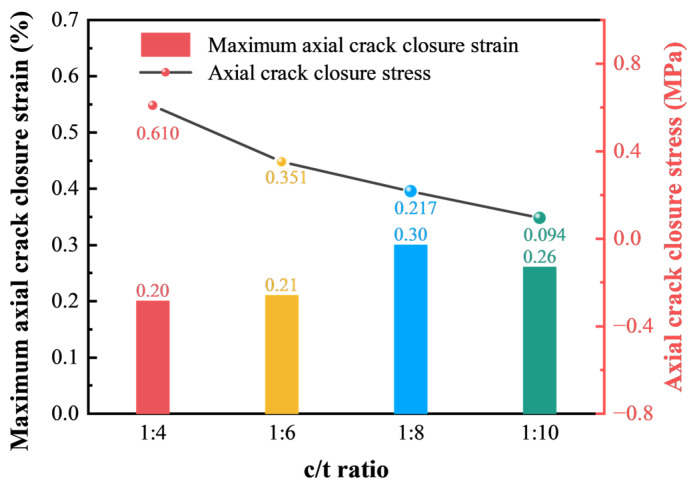
Relationship between maximum axial crack closure strain, closure stress, and the c/t ratio.

Figure 16 demonstrates the relationship between axial crack strain (maximum axial crack closure strain), the corresponding stress at the initial crack closure point (axial crack closure stress), and different c/t ratios. The maximum axial crack closure strain reflects the influence of complete closure of initial microdefects in CTB on axial strain, where a higher value indicates a greater initial microdefect content. As shown in Figure 16, the maximum axial crack closure strain increases with the decreasing c/t ratio. Among all c/t ratios, a c/t ratio of 1:8 exhibits the highest maximum axial crack strain at 0.30%, indicating the highest initial microdefects content. Conversely, the axial stress at the initial crack closure point falls as the c/t ratio decreases. It reaches a maximum of 0.610 MPa for a c/t ratio of 1:4 and a minimum of 0.094 MPa for a c/t ratio of 1:10, because a higher c/t ratio enhances CTB strength, requiring greater stress for the closure of microdefects.

**Figure 17:**
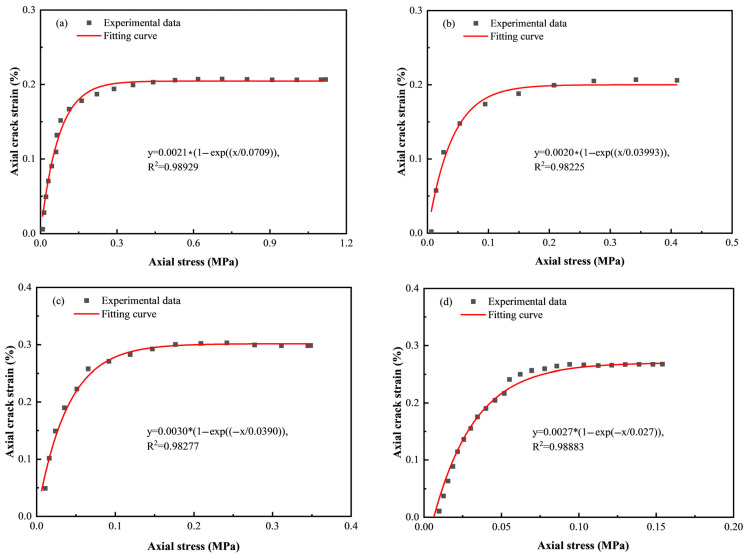
Relationship between axial crack strain and axial stress: (**a**) c/t = 1:4; (**b**) c/t = 1:6; (**c**) c/t = 1:8; (**d**) c/t = 1:10.

The axial crack strain from initially and newly generated microdefects starts at zero and remains constant after the compaction stage as the axial loading increases. The axial crack strain–axial stress curves are fitted with negative exponential functions, as presented in Figure 17. The *R*^2^ values are 0.98929, 0.98225, 0.98277 and 0.98883, corresponding to c/t ratios of 1:4, 1:6, 1:8, and 1:10, respectively. Accordingly, in the initial compaction stage, the axial crack stain follows a negative exponential function as axial stress increases.

## 4. Construction of the Damage Constitutive Model of CTB Samples

### 4.1. Construction of the Damage Constitutive Model Considering the Initial Compaction Stage

According to Lemaitre’s equivalent strain principle [55,56], assuming the CTB sample is isotropic, the classic damage constitutive model of CTB can be formulated as follows:

(9) $$\sigma_{1}=E\varepsilon_{1}\left(1-D\right)$$

where *D* is the damage variable.

Based on the phenomenological statistical meso-mechanics, the macroscopic failure of CTB can be regarded as the average effect of numerous meso-element failures. Therefore, different expressions for the damage variable can be attained by employing different probability distribution functions to represent the failure of material meso-elements [57]. In this paper, assuming that the load damage of CTB micro-elements follows the Weibull distribution, namely:

(10) $$P\left(n\right)=\frac{m}{n}{\left(\frac{\varepsilon }{n}\right)}^{m-1}\exp\left[-{\left(\frac{\varepsilon }{n}\right)}^{m}\right]$$

where $P\left(n\right)$ is the meso-element strength distribution function of CTB, and *m* and *n* are distribution parameters of the Weibull distribution.

When the strain of CTB reaches $\varepsilon_{1}$ under loading, the damage value is as follows:

(11) $$D=1-\exp\left[-{\left(\frac{\varepsilon_{1}}{n}\right)}^{m}\right]$$

When D=0, there is no damage in CTB; when D=1, CTB is completely damaged.

Combining Equation (9) with Equation (11), the damage variable can be formulated as:

(12) $$\sigma_{1}=E\varepsilon_{1}\exp\left[-{\left(\frac{\varepsilon_{1}}{n}\right)}^{m}\right]$$

However, Equation (9) does not take initial microdefects into consideration and can only characterize the stress–strain evolution of CTB without initial microdefects.

To address the problem, the CTB sample with microdefects can be regarded as a sum of one intact portion without microdefects and the other portion contains only microdefects. The strain of the intact portion of CTB that can undergo damage can be obtained:

(13) $${\varepsilon_{1}}^{m}=\varepsilon_{1}-{\varepsilon_{1}}^{c}$$

where ${\varepsilon_{1}}^{c}$ is the strain of the portion containing only microdefects, and ${\varepsilon_{1}}^{m}$ represents the strain of the intact portion without microdefects.

Substituting ${\varepsilon_{1}}^{m}$ for $\varepsilon_{1}$, a modified constitutive model that considers the influence of initial microdefects can be obtained:

(14) $$\sigma_{1}=E{\varepsilon_{1}}^{m}\left(1-D\right)$$

As shown in Figure 15 and Figure 17, during the compaction stage, axial crack strain is correlated with axial stress, and can be described by a negative exponential function as follows:

(15) $${\varepsilon_{1}}^{c}=\left\{\begin{array}{cc} a\left(1-\exp\left(-\frac{\sigma_{1}}{b}\right)\right) & \varepsilon_{1}<\varepsilon_{0}\\ {\varepsilon_{0}}^{c} & \varepsilon_{1}>\varepsilon_{0} \end{array}\right.$$

where *a* and *b* are fitting parameters, ${\varepsilon_{0}}^{c}$ is the axial crack strain at the initial microcrack closure point (onset of the elastic stage), and $\varepsilon_{0}$ is the axial strain at the initial microcrack closure point, corresponding to turning point A in Figure 15.

Inserting Equation (13) into Equation (15), the following formula can be derived:

(16) $$\left\{\begin{array}{cc} \varepsilon_{1}=\frac{\sigma_{1}}{E\left(1-D\right)}+a\left(1-\exp\left(-\frac{\sigma_{1}}{b}\right)\right) & \varepsilon_{1}<\varepsilon_{0}\\ \sigma_{1}=E\left(\varepsilon_{1}-{\varepsilon_{0}}^{c}\right)\left(1-D\right) & \varepsilon_{1}>\varepsilon_{0} \end{array}\right.$$

During the compaction stage, the axial stress of CTB under external load is much smaller than the yield strength, and the intact sample does not damage. From an energy perspective, the input energy of CTB accumulates slowly, meaning the damage variable remains zero, and the intact portion of it can still undergo damage. Therefore, the axial strain in Equation (13) could be replaced by ${\varepsilon_{1}}^{m}$ and the expression for the modified damage variable is derived:

(17) $$D=\left\{\begin{array}{cc} 0 & \varepsilon_{1}<\varepsilon_{0}\\ 1-\exp\left(-{\left(\frac{\varepsilon_{1}-{\varepsilon_{0}}^{c}}{n}\right)}^{m}\right) & \varepsilon_{1}>\varepsilon_{0} \end{array}\right.$$

Substituting Equation (17) into Equation (16), the complete modified constitutive model can be obtained:

(18) $$\left\{\begin{array}{cc} \varepsilon_{1}=\frac{\sigma_{1}}{E}+a\left(1-\exp\left(-\frac{\sigma_{1}}{b}\right)\right) & \varepsilon_{1}<\varepsilon_{0}\\ \sigma_{1}=E\left(\varepsilon_{1}-{\varepsilon_{0}}^{c}\right)\exp\left(-{\left(\frac{\varepsilon_{1}-{\varepsilon_{0}}^{c}}{n}\right)}^{m}\right) & \varepsilon_{1}>\varepsilon_{0} \end{array}\right.$$

### 4.2. Determination of the Constitutive Model Parameters of CTB

(1) Determination of a and b

When $\varepsilon_{1}<\varepsilon_{0}$, parameters *a* and *b* can be acquired by fitting the negative exponential curve of the axial crack strain–axial stress. The fitting results, as shown in Figure 17, are listed in Table 3.

(2) Determination of *m*, *n* and ${\varepsilon_{0}}^{c}$

(19) $$\left\{\begin{array}{c} \sigma_{1}{|}_{\varepsilon_{1}={\varepsilon_{1}}^{p}}={\sigma_{1}}^{p}\\ {\frac{d\sigma_{1}}{d\varepsilon_{1}}|}_{\varepsilon_{1}={\varepsilon_{1}}^{p}}=0 \end{array}\right.$$

where ${\varepsilon_{1}}^{p}$ and ${\sigma_{1}}^{p}$ represent the peak strain and the corresponding peak strength.

${\varepsilon_{0}}^{c}$ can be obtained in Figure 16. Except ${\varepsilon_{0}}^{c}$, there are two unknown variables in Equation (19). Solving Equation (19) yields the parameters *m* and *n* as follows:

(20) $$\left\{\begin{array}{c} m=-1/\ln\left[\frac{{\sigma_{1}}^{p}}{E\left({\varepsilon_{1}}^{p}-{\varepsilon_{0}}^{c}\right)}\right]\\ n=\frac{\left({\varepsilon_{1}}^{p}-{\varepsilon_{0}}^{c}\right)}{\sqrt[{m}]{1/m}} \end{array}\right.$$

The parameters *m*, *n* and ${\varepsilon_{0}}^{c}$ of CTB with different c/t ratios are listed in Table 4.

(3) The parameterized constitutive model for CTB

According to fitting results in Figure 7, Figure 8 and Figure 9 in Section 3.1, the elasticity modulus, peak strain, and peak strength of CTB have exponential relationships with the c/t ratio, namely:

(21) $$E=21.779\exp\left(\frac{x}{0.082}\right)-47.869$$

(22) $${\varepsilon_{1}}^{p}=11.423\exp\left(-\frac{x}{0.041}\right)+0.971$$

(23) $${\sigma_{1}}^{p}=0.558\exp\left(\frac{x}{0.156}\right)-0.784$$

Considering the effect of different c/t ratios on the constitutive model, the constitutive model is further modified by substituting Equations (20)–(23) into Equation (18), yielding:

(24) $$\left\{\begin{array}{cc} \varepsilon_{1}=\frac{\sigma_{1}}{E\left(x\right)}+a\left(1-\exp\left(-\frac{\sigma_{1}}{b}\right)\right) & \varepsilon_{1}<\varepsilon_{0}\\ \sigma_{1}=E\left(x\right)\left(\varepsilon_{1}-{\varepsilon_{0}}^{c}\right)\exp\left[-{\left(\frac{\varepsilon_{1}-{\varepsilon_{0}}^{c}}{n\left(x\right)}\right)}^{m\left(x\right)}\right] & \varepsilon_{1}>\varepsilon_{0} \end{array}\right.$$

The modified constitutive model not only considers the influence of initial microdefects in CTB but also parameterizes the parameters of CTB. Therefore, a new dependent variable *x* was introduced to the constitutive model, and the functional relationship between axial stress, axial strain, and the c/t ratio is expressed as $\sigma_{1}=f\left(\varepsilon_{1},x\right)$.

### 4.3. Validation of the Proposed Damage Constitutive Model of CTB

The developed constitutive model incorporates the compaction characteristics of CTB, with the unknown material parameters determined within the model.

(1) Validation of the damage constitutive model in the initial compaction stage

**Figure 18:**
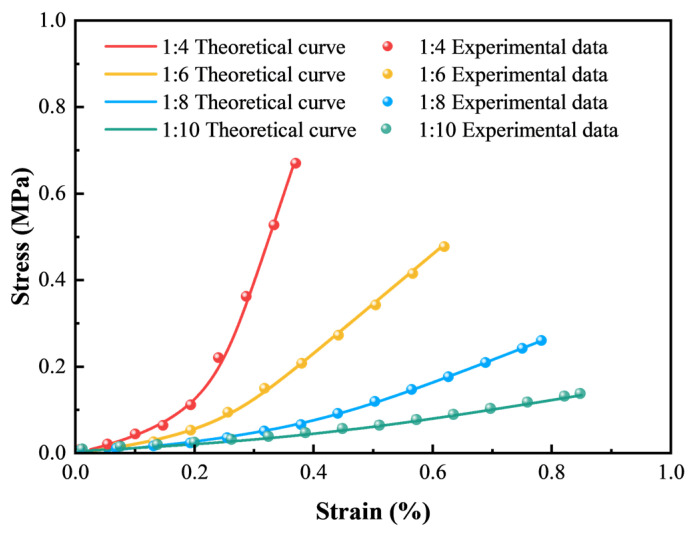
Comparison of theorical curves and experimental data in the compaction stage.

The theoretical stress–strain curves based on the proposed constitutive model for CTB with different c/t ratios in the initial compaction stage are compared with experimental data in Figure 18. The results indicate that the theoretical curves closely agree with experimental data.

(2) Validation of the complete damage constitutive model

**Figure 19:**
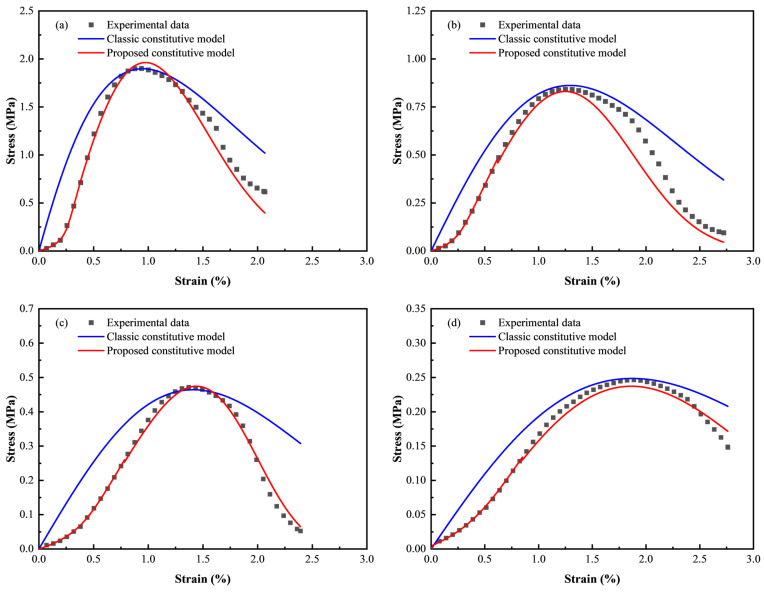
Comparison of theorical curves and experimental data: (**a**) c/t = 1:4; (**b**) c/t = 1:6; (**c**) c/t = 1:8; (**d**) c/t = 1:10.

Starting from the theoretical curve in the initial compaction stage, the complete theoretical curve is derived by integrating the constitutive equation for subsequent stages. To further substantiate the proposed constitutive model, theoretical curves derived from it and classic damage constitutive (Equation (17)) are compared with experimental data of CTB with varying c/t ratios, as illustrated in Figure 19. Specifically, the curves based on the proposed theoretical model closely match experimental data, especially before the peak points. The fitting results of the initial compaction, elastic, and plastic stages are remarkably better than those of the classical model. Without considering the influence of initial microdefects, the classical constitutive model curve directly begins with the elastic stage, leading to deviations in the compaction and plastic stages. Hence, the classical model is more suitable for materials with few or no initial microdefects, while the proposed model better conforms to the stress and deformation behaviors of actual CTB.

### 4.4. Energy Matching of CTB with Surrounding Rocks

The filling body is vital for controlling ground pressure and supporting roof rock. After filling, CTB restricts rock deformation, absorbs energy by deforming under compression from roof and surrounding rocks, facilitating energy transfer and ensuring mining safety.

#### 4.4.1. Energy Dispassion Characteristics of Surrounding Rock Excavation

Because the rock is in the elastic phase before excavation, surrounding rocks can be analyzed using a linear elastic model, namely:

(25) $$\sigma_{R}=E_{R}\varepsilon_{R}$$

where $\sigma_{R}$, $E_{R}$ and $\varepsilon_{R}$ are the stress, the elasticity modulus and the strain of surrounding rocks, respectively.

The stress of surrounding rocks is gravity stress, without considering the tectonic stress. Therefore, after mining, the stress is $\sigma_{R}=\gamma H$, where γ and *H* are the unit weight and the burial depth of rock mass. Considering a one-dimensional stress state, the strain energy released per unit volume after the excavation of the surrounding rock is formulated:

(26) $$U_{R}=\int_{0}^{\varepsilon_{0}}\sigma_{R}d\varepsilon_{R}=\frac{\sigma_{R}^{2}}{2E_{R}}=\frac{\gamma^{2}h^{2}}{2E_{R}}$$

#### 4.4.2. Deformation Energy Absorbed by CTB

According to the pre-peak portion of the established constitutive model (Equation (18)) in Section 4.1, the energy absorbed by per unit backfill under compression is derived:

(27) $$U_{P}=\int_{0}^{{\varepsilon_{1}}^{p}}\sigma_{1}d\varepsilon_{1}$$

**Figure 20:**
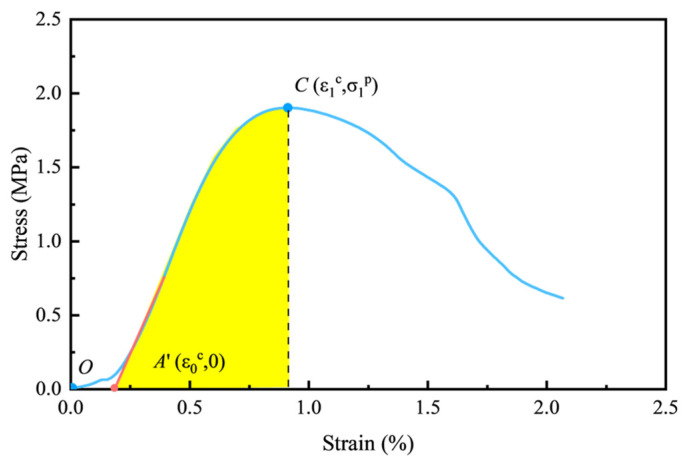
Deformation energy calculation of CTB.

The proposed constitutive model during the compaction stage shows that strain is implicitly related to stress, complicating integration. To facilitate engineering applications, the strain of the intersection point (*A*’) of the stress–strain curve’s elastic stage and the strain axis is determined as the initial compaction strain. As illustrated in Figure 20, the area shaded yellow approximates the peak strain energy of CTB. Although the energy calculated is slightly lower than the actual energy, the conservative calculation ensures increased safety and reliability in engineering.

(28) $$U_{P}=\int_{{\varepsilon_{0}}^{c}}^{{\varepsilon_{1}}^{p}}E(\varepsilon_{1}-{\varepsilon_{0}}^{c})\exp\left[-{\left(\frac{\varepsilon_{1}-{\varepsilon_{0}}^{c}}{n}\right)}^{m}\right]d\varepsilon =\int_{0}^{{\varepsilon_{1}}^{p}-{\varepsilon_{0}}^{c}}Ex\exp\left[-{\left(\frac{x}{n}\right)}^{m}\right]dx$$

Because *m* might not be an integer, direct integration of the function is not feasible, so we expand the exponential function using a Taylor series, yielding:

(29) $$e^{x}=\sum_{l=0}^{\infty }\frac{x^{l}}{l!}$$

The larger the value of *l*, the more terms in the Taylor series expansion, and the closer the result is to the actual value. When *l* equals 3, the exponential function is expanded using a Taylor series, we obtain an integral of a polynomial function:

(30) $$U_{P}=\int_{0}^{{\varepsilon_{1}}^{p}-{\varepsilon_{0}}^{c}}Ex(1-{\left(\frac{x}{n}\right)}^{m}+\frac{1}{2}{\left(\frac{x}{n}\right)}^{m^{2}}-\frac{1}{6}{\left(\frac{x}{n}\right)}^{m^{3}}+o(x^{m^{3}}))dx$$

where $o(x^{m^{3}})$ is the Peano remainder term.

Given that the magnitude of *x* is 10^−2^ and *m* is approximately 2, when *m* is set to 2, the magnitude of $x^{m^{3}}$ is 10^−16^, which is virtually zero. This term can be completely neglected in engineering and can be replaced by 0. Moreover, the last term in the expansion formula is negative, which means the calculated result will be slightly smaller than the actual value, but it is within the error allowed and thus meets engineering requirements. When stress of CTB reaches its peak, the absorbed strain energy is obtained:

(31) $$U_{P}=E[\frac{{\left({\varepsilon_{1}}^{p}-{\varepsilon_{0}}^{c}\right)}^{2}}{2}-\frac{{\left({\varepsilon_{1}}^{p}-{\varepsilon_{0}}^{c}\right)}^{m+2}}{n^{m}(m+2)}+\frac{{\left({\varepsilon_{1}}^{p}-{\varepsilon_{0}}^{c}\right)}^{m^{2}+2}}{2n^{m^{2}}(m^{2}+2)}-\frac{{\left({\varepsilon_{1}}^{p}-{\varepsilon_{0}}^{c}\right)}^{m^{3}+2}}{6n^{m^{3}}(m^{3}+2)}]$$

Considering the principle that the energy released from the rock mass is nearly equal to the peak deformation energy of CTB, the matching coefficient *K* as the ratio of the *U*_p_ to *U*_R_ is derived [58]:

(32) $$K=\frac{U_{P}}{U_{R}}=\frac{2EE_{R}}{{\sigma_{V}}^{2}}[\frac{{\left({\varepsilon_{1}}^{p}-{\varepsilon_{0}}^{c}\right)}^{2}}{2}-\frac{{\left({\varepsilon_{1}}^{p}-{\varepsilon_{0}}^{c}\right)}^{m+2}}{n^{m}(m+2)}+\frac{{\left({\varepsilon_{1}}^{p}-{\varepsilon_{0}}^{c}\right)}^{m^{2}+2}}{2n^{m^{2}}(m^{2}+2)}-\frac{{\left({\varepsilon_{1}}^{p}-{\varepsilon_{0}}^{c}\right)}^{m^{3}+2}}{6n^{m^{3}}(m^{3}+2)}]$$

When *K* > 1, the deformation energy of CTB is sufficient to support the energy released by surrounding rocks, preventing energy instability and ensuring a match with surrounding rocks. Conversely, when *K* < 1, the energy in the CTB becomes unstable, indicating a mismatch with surrounding rocks.

#### 4.4.3. Engineering Background

The 5# ore body of Chaihulanzi Gold Mine spans depths of 141 to 390 m with an average dip greater than 70°, characterizing it as a steeply inclined, fragmented thin ore body. The elastic moduli of the footwall, ore body, hanging wall, and bedrock are 12.76 GPa, 15.83 GPa, 16.55 GPa, and 14.66 GPa, respectively. We select 16.55 GPa as the modulus for the overlying strata and 27 kN/m^3^ for unit weight. According to geostress measurement results, the vertical geostress is:

(33) $$\sigma_{V}=0.02901H$$

**Figure 21:**
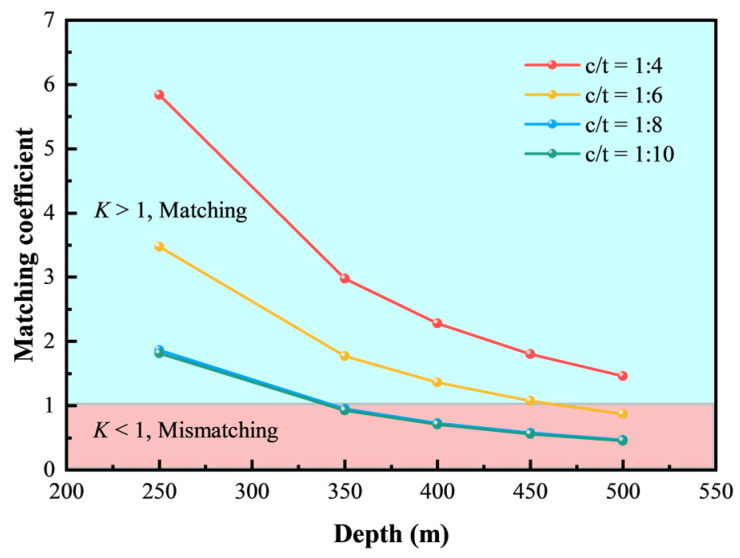
Matching coefficients of CTB and surrounding rocks at different depths.

Energy matching between CTB and surrounding rocks was conducted for overburden thicknesses of 250 m, 350 m, 400 m, 450 m, and 500 m, with the values of *K* shown in Figure 21. Figure 21 indicates that *K* decreases as the depth increases and the c/t ratio decreases, with slight differences between 1:8 and 1:10. For current mining depths (less than 400 m), CTB with c/t ratios of 1:6 and 1:4 can match the energy released by surrounding rocks, making them appropriate for all existing mining levels. For mining depths shallower than 300 m, CTB with a c/t ratio of 1:10 is sufficient to maintain stope stability. Taking a 400 m overlying rock mass as an example, CTB with c/t ratios of 1:4 and 1:6 can adequately match surrounding rocks, whereas other ratios may cause instability. However, economically speaking, a 1:6 c/t ratio is optimal. With the prospect of new ore bodies and rising mining depths, CTB fails to match rock mass effectively. Therefore, enhancing the cement material proportion or improving the slurry concentration is required to optimize CTB mechanical properties and match deep rock mass.

## 5. Conclusions

The macroscopic mechanical properties, stress–strain curves, macroscopic failure modes, microcrack distribution characteristics, and energy evolution patterns of CTB under uniaxial compression were systematically investigated with c/t ratios of 1:4, 1:6, 1:8, 1:10, and a fixed mass fraction of 78%. A constitutive model considering the initial compaction stage was developed, validated and applied based on the negative exponential relationship between axial crack strain and axial stress. The following conclusions can be obtained:

(1) The effects of c/t ratio on the mechanical properties of CTB follow exponential functions, exhibiting higher *R*^2^ values compared to polynomial functions. The peak stress, the residual stress, and the elasticity modulus of CTB decrease, while the peak strain increases with the decreasing c/t ratio.
(2) Macroscopically, CTB with different c/t ratios presents diverse failure modes. As the c/t ratio increases, the failure mode transforms from line-shaped tensile failure to combined tension–shear failure and diagonal shear failure. Microscopically, the total microcrack number and shear microcrack number increase consequently, whereas the tension microcrack number decreases with increasing c/t ratios.
(3) The energy evolution of CTB with different c/t ratios follows similar trends, progressing through the stages of initial compaction, elastic, plastic, and failure. The elastic strain energy increases slowly at first, then dramatically, and finally decreases gradually with axial strain increasing, and the dissipated energy increases in a “gentle–rapid–steady–slow” pattern.
(4) A precise damage constitutive model that considers the initial compaction stage and axial crack strain evolution was developed. The theoretical curves match well with experimental data, accurately reflecting the mechanical parameters and damage characteristics of CTB with varying c/t ratios.
(5) The matching coefficient *K*, representing the ratio of the peak deformation energy absorbed by CTB to energy dispassion of surrounding rock excavation, was calculated to assess the reasonable matching in the 5# ore body of Chaihulanzi Gold Mine. Economically and safely, a 1:10 c/t ratio is optimal to mining depths under 300 m, while a 1:6 c/t ratio is adequate for all current mining levels.

## Figures and Tables

**Table 1:** The *R*^2^ values of fitting between mechanical parameters and the c/t ratio of CTB.

*R* ^2^	Peak Stress (MPa)	Residual Stress (MPa)	Elasticity Modulus (GPa)	Peak Strain (%)	Mean (%)
Polynomial function fitting	0.99978	0.98445	0.99867	0.93009	0.97825
Exponential function fitting	0.99991	0.98739	0.99994	0.96233	0.98739

**Table 2:** Meso-parameters of CTB in simulation.

	Parameters	1:4	1:6	1:8	1:10
Tailings particles	Effective modulus (MPa)	600			
	Normal-to-shear stiffness ratio	1.5			
	Density (kg/m^3^)	2670			
	Radius (m)	1–1.5 × 10^−4^			
	Initial void ratio	0.3			
Cement particles	Effective modulus (MPa)	220	84	50	20
	Normal-to-shear stiffness ratio	1.5	1.5	1.4	1.4
	Radius (m)	1 × 10^−4^			
	Density (kg/m^3^)	2500			
Total number of particles		22,575	20,586	19,392	18,596
Parallel bond contact	Bond effective modulus (MPa)	328	100	30	20
	Bond normal-to-shear stiffness ratio	1.5	1.5	1.4	1.4
	Cohesion (MPa)	4.26	2.45	1.71	1.02
	Tensile strength (MPa)	8.52	3.93	2.06	0.82
	Friction angle (°)	50	50	50	50
	Friction coefficient	0.5	0.47	0.43	0.40

**Table 3:** Fitting parameters when $\varepsilon_{1}<\varepsilon_{0}$.

c/t	*a*	*b*	Constitutive Model	*R* ^2^
1:4	0.0021	0.0709	$\varepsilon_{1}=0.0021\left(1-\exp\left(-\sigma_{1}/0.0709\right)\right)$	0.98929
1:6	0.0020	0.0399	$\varepsilon_{1}=0.0020\left(1-\exp\left(-\sigma_{1}/0.0399\right)\right)$	0.98225
1:8	0.0030	0.0390	$\varepsilon_{1}=0.0030\left(1-\exp\left(-\sigma_{1}/0.0399\right)\right)$	0.98277
1:10	0.0027	0.0270	$\varepsilon_{1}=0.0027\left(1-\exp\left(-\sigma_{1}/0.0270\right)\right)$	0.98883

**Table 4:** Fitting parameters when $\varepsilon_{1}>\varepsilon_{0}$.

c/t	*m*	*n*	${\varepsilon_{0}}^{c}$/%	Constitutive Model
1:4	2.056	0.011	0.20	$\sigma_{1}=E\left(\varepsilon_{1}-{\varepsilon_{0}}^{c}\right)\exp\left[-{\left(\left(\varepsilon_{1}-{\varepsilon_{0}}^{c}\right)/0.011\right)}^{2.056}\right]$
1:6	2.756	0.015	0.21	$\sigma_{1}=E\left(\varepsilon_{1}-{\varepsilon_{0}}^{c}\right)\exp\left[-{\left(\left(\varepsilon_{1}-{\varepsilon_{0}}^{c}\right)/0.015\right)}^{2.756}\right]$
1:8	3.966	0.016	0.30	$\sigma_{1}=E\left(\varepsilon_{1}-{\varepsilon_{0}}^{c}\right)\exp\left[-{\left(\left(\varepsilon_{1}-{\varepsilon_{0}}^{c}\right)/0.016\right)}^{3.966}\right]$
1:10	2.266	0.023	0.27	$\sigma_{1}=E\left(\varepsilon_{1}-{\varepsilon_{0}}^{c}\right)\exp\left[-{\left(\left(\varepsilon_{1}-{\varepsilon_{0}}^{c}\right)/0.023\right)}^{2.266}\right]$

## Data Availability

The original contributions presented in this study are included in the article. Further inquiries can be directed to the corresponding author.

## Nomenclature

CPB	Cemented paste backfill	PBM	Parallel bond contact model
c/t	Cement to tailings	SEM	Scanning electron microscope
CTB	Cemented tailings backfill	UCS	Uniaxial compressive strength
DFN	Discrete fracture network	XRF	X-ray fluorescence
PFC	Particle Flow Code		
*a*, *b* [-]	Fitting parameters in Equation (15)	$\varepsilon_{0}$ [-]	Axial strain at initial microcrack closure point
*D* [-]	Damage variable	${\varepsilon_{0}}^{c}$ [-]	Axial crack strain at initial microcrack closure point
*E* [Pa]	Elasticity modulus of CTB	$\varepsilon_{1}$ [-]	Axial strain of CTB
$E_{R}$ [Pa]	Elasticity modulus of rock mass	${\varepsilon_{1}}^{c}$ [-]	Axial crack strain of CTB
*H* [m]	Burial depth of rock mass	${\varepsilon_{1}}^{m}$ [-]	Strain of CTB of the intact portion without microdefects
*K* [-]	Matching coefficient	$\varepsilon_{1}^{p}$ [-]	Peak strain of CTB
*m*, *n* [-]	Distribution parameters of the Weibull distribution in Equation (10)	$\varepsilon_{R}$ [-]	Axial strain of rock mass
$P\left(n\right)$ [-]	Meso-element strength distribution function	γ [N/m^3^]	Unit weight of rock mass
U [J]	Total energy	$\sigma_{1}$ [Pa]	Axial stress of CTB
$U_{e}$ [J]	Elastic strain energy	$\sigma_{R}$ [Pa]	Axial stress of rock mass
$U_{d}$ [J]	Dissipated energy	$\sigma_{r}$ [Pa]	Residual stress of CTB
$U_{P}$ [J]	Peak strain energy of CTB	$\sigma_{p}$ [Pa]	Peak strength of CTB
$U_{R}$ [J]	Peak strain energy of rock mass	$\sigma_{V}$ [Pa]	Vertical geostress
*x* [-]	c/t ratio		
